# Supplementary material for: Neural structure of a sensory decoder for motor control
Source: Nat Commun. 2022 Apr 5;13:1829. doi: 10.1038/s41467-022-29457-4 (PMC8983777; doi:10.1038/s41467-022-29457-4)
Supplement: Supplementary file 1 — Supplementary Information [file 41467_2022_29457_MOESM1_ESM.pdf]

Supplementary Information

Neural structure of a sensory decoder for motor control

Seth W. Egger and Stephen G. Lisberger

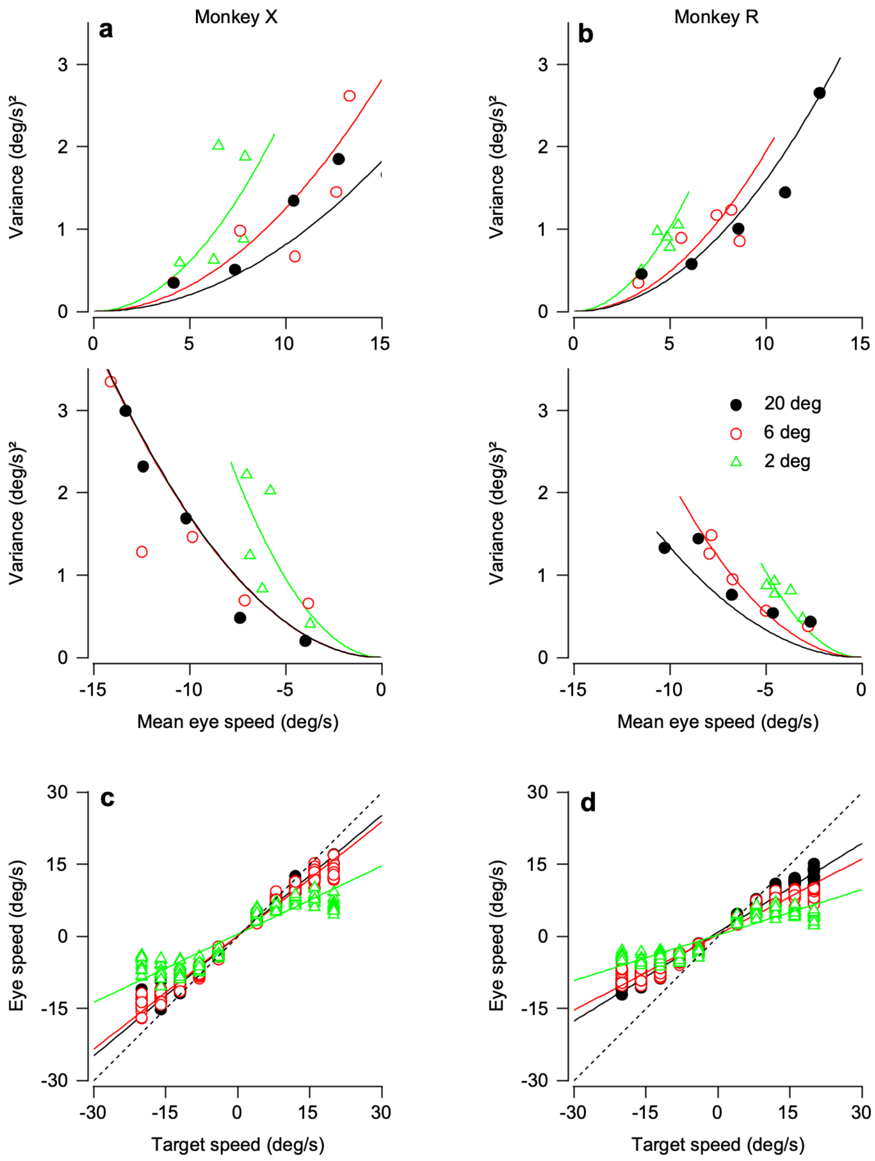

Supplementary Figure 1. Effect of fixation eccentricity at motion onset on variability and gain of pursuit responses to each target size. To assess the degree to which variation in fixation position at target onset influenced our results, we reanalyzed our data using trials with fixation within 2.5 and 2 deg of the fixation point for monkeys X and R, respectively. a) Variance in pursuit initiation by monkey X as a function of mean eye speed for right pursuit (top) and left pursuit (bottom). Green triangles, red circles, and black circles correspond to the data for the small, medium, and large targets, respectively. b) As in panel a, but for monkey R. c) Trial-by-trial eye speed as a function of target speed for monkey X. Dashed line plots unity. Solid lines plot the best fitting linear model for each target condition. Color and marker conventions as in panel a. d) As in panel c, but for monkey R. The analysis shows that our behavioral results do not critically depend on the degree to which the monkeys accurately fixated the target at motion onset. Source data are provided as a Source Data file.

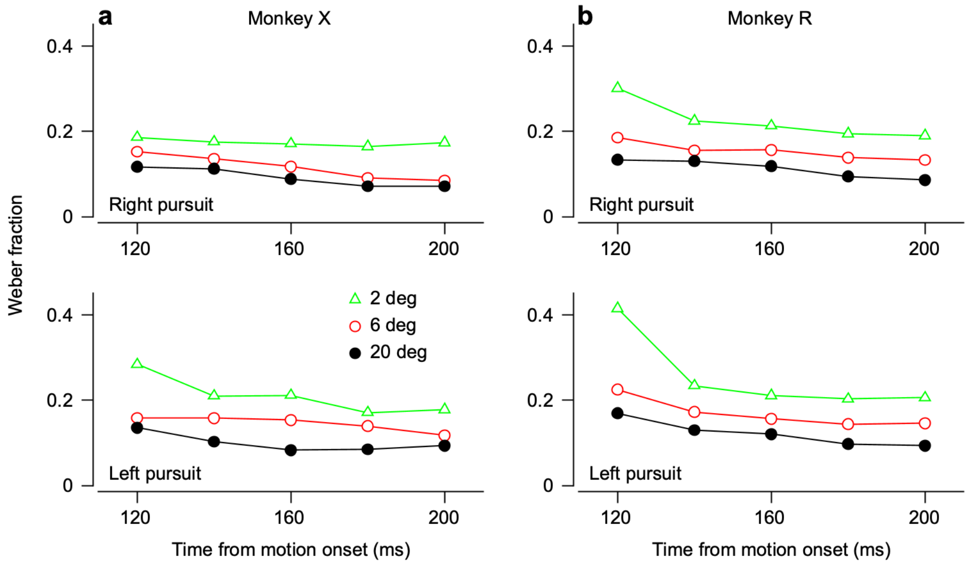

Supplementary Figure 2. Target size affects Weber fractions across the full interval of the initiation of pursuit. We divided the interval from 120 to 200 ms after the onset of target motion into intervals of duration 20 ms and analyzed the Weber fraction for each target size in each interval. Green, red, and black symbols show data for target sizes of 2, 6, and 20 deg and indicate that the Weber fraction is consistently smaller for larger targets across the entire interval of pursuit initiation. Panels a and b plot the data for monkeys X and R, respectively. Source data are provided as a Source Data file.

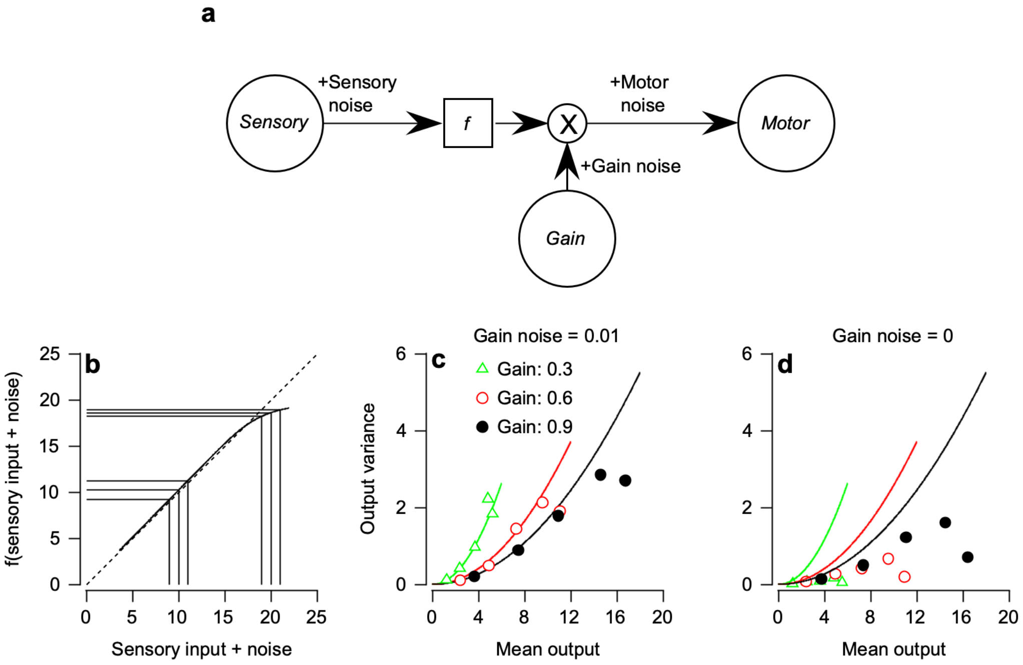

Supplementary Figure 3. Generalization of the gain-noise model to nonlinear sensory encoding and estimation. To examine whether the signature of gain noise could result from other changes in model configuration, we generalized the simple model in Figure 3 to include a nonlinear transformation. In panel a, a sensory input, s, with signal-dependent noise, η_s, is rendered non-linear according to the function f. A gain signal with mean G and variance σ^2 is applied following the transformation by f, and motor noise added following the gain. For small values of η_s, the variance will be:

$$\mathrm{VAR} = w_s^2 G^2 s^2 \left(\frac{df}{ds}\bigg|_s\right)^2 + \sigma^2 w_s^2 s^2 \left(\frac{df}{ds}\bigg|_s\right)^2 + \left(w_m + \frac{\sigma^2}{G^2}\right)\mu^2,$$

where w_s is the Weber fraction that determines the magnitude of sensory noise according to target speed ($\sigma_s^2 = w_s^2 s^2$), w_m is the motor Weber fraction that determines the magnitude of motor noise according to $\sigma_m^2 = w_m^2 \mu^2$, and μ is the mean output of the model. The first two terms of the equation cause the variance of motor output to depend on the derivative of f, df/ds, in the vicinity of s (see Supplementary Note 2). The last term of the equation causes the output variance to depend on the mean gain when gain noise is non-zero. Panel b illustrates the effect of a nonlinear transformation, f, based on the Bayesian least squares (BLS) estimator for signal-dependent sensory noise ($w = 0.1$)[1–3]. In the vicinity of $s + \eta_s = 10$, df/ds is near 1. As a result, sensory noise illustrated by the 3 parallel vertical lines near $s + \eta_s = 10$, adds variance to the output of f, as shown by the three horizontal lines that project the sensory input onto the y-axis. In the vicinity of $s + \eta_s = 20$, df/ds starts to approach 0. Accordingly, the same amount of sensory noise shown by the vertical lines near $s + \eta_s = 20$ leads to very little output variance, shown by the projection onto the y-axis (horizontal lines). Panels c and d plot the output variance as a function of the mean output amplitude for different configurations of the model and illustrate that a plausible non-linearity alone is not enough to produce the predictions of gain noise. Panel c reproduces Figure 3 with gain set to 0.3 (green), 0.6 (red), or 0.9 (black) and the gain noise set to have a variance of 0.01 (standard deviation of 0.1). The continuous curves show the best fits to the model outputs based on the model in Figure 3. Panel d illustrates the performance of the non-linear model with gain noise set to zero and shows that the nonlinearity does not convey the same effect as gain noise. Output variance is still signal-dependent, but the data do not separate according to the value of gain. Note that, while the nonlinearity should not affect the results for target speed in the linear range of f, it might (but does not) cause the signature of gain noise for the highest values of target speed. The continuous curves in panel d are the same as those in panel c, for easy visual comparison of the performance of different configurations of the model. Source data are provided as a Source Data file.

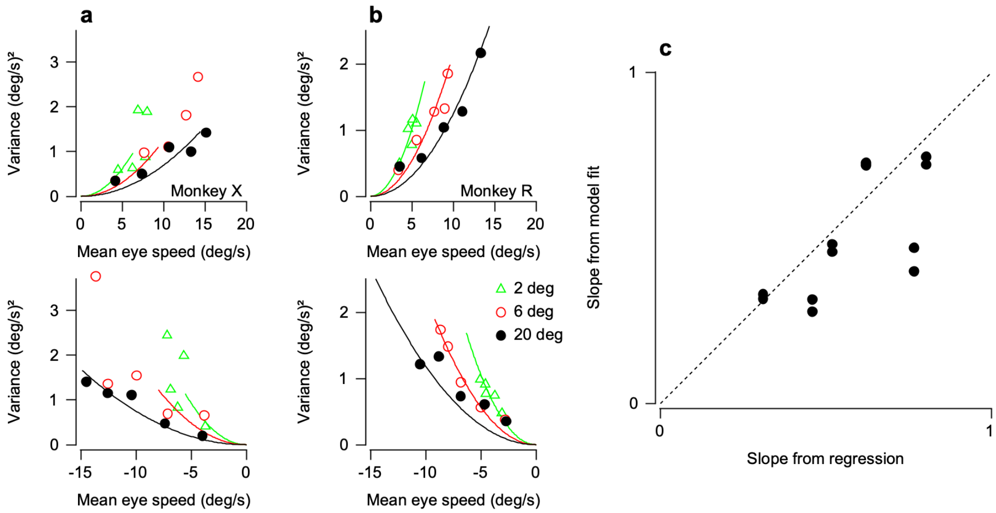

Supplementary Figure 4. Comparison of gain inferred by regression to the gain inferred from the relationship between the mean and variance of pursuit responses. Our analysis in Figure 2 provided an estimate of the gain for each size condition through regression. Equation (1) implies that we can also infer the gain from the relationship between the mean and variation in motor responses. We therefore fit the gain-noise model using Equation (4), but allowed G to change as a function of the stimulus size. Panels a and b show the fit of the model to the mean and variance of pursuit responses by monkey X and R, respectively, to rightward (top) and leftward trials (bottom). Conventions as in Figure 1. We then compare the gains inferred from the noise model to that inferred from regression (i.e. Figure 2b and c) in panel c. There is substantial agreement between the two methods for inferring the gain. It should be noted, however, that an exact agreement should not be expected. Our gain-noise model is based on the estimated speed, $\hat{s}$, which can be nonlinear with respect to speed, s. The gain inferred from regression, however, assumes linearity in estimation. Therefore, an exact agreement would only be expected if the monkeys linearly estimated speed (see Supplementary Note 2). Figure 2b and c suggest the assumption of linearity is not correct. Source data are provided as a Source Data file.

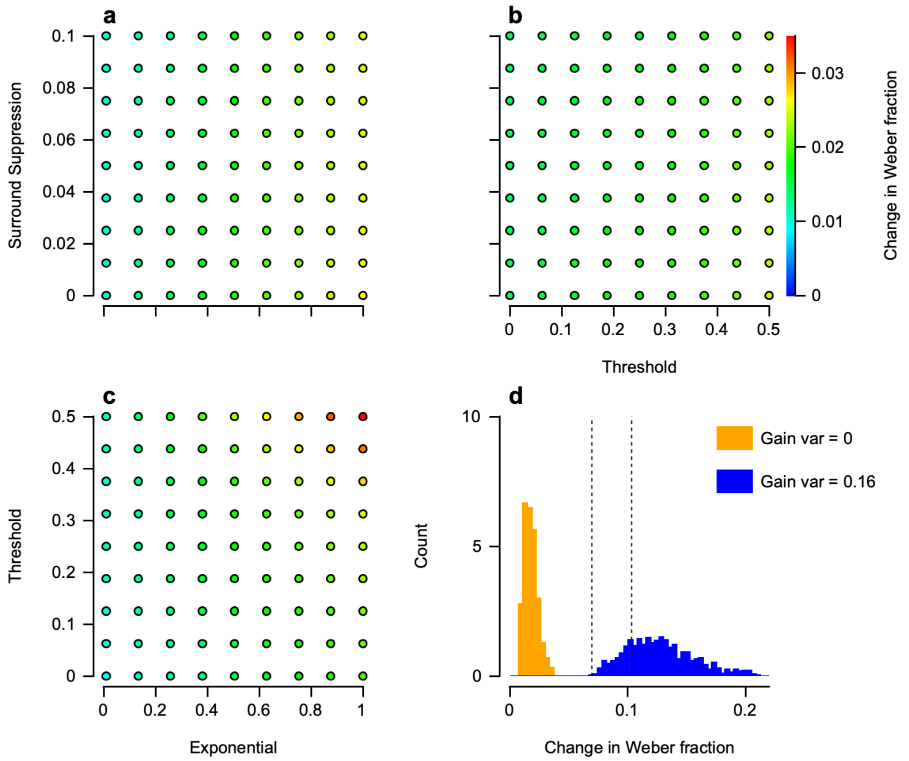

Supplementary Figure 5. Gain noise is required to match behavior across a range of circuit model parameterizations. To determine the degree to which the parameters of MT model neurons can replicate behavioral results without gain noise, we measured circuit model behavior while systematically varying each parameter. For each model simulation, we used a specific combination of the exponential (n), surround suppression (β), and threshold (ω) of MT model neurons (see Methods). Across model simulations, we systematically sampled each possible parameter combination. For each simulated parameter combination, we fit a model of the form $\sigma^2 = w^2\mu^2$ to the mean and variance of the circuit output, and allowed w to change with target size. Panel a plots the change in Weber fraction, measured as the difference in w fit to the 2 deg target and w fit to the 20 deg target (colors; see color bar in panel b), for each combination of exponential and surround suppression, averaged across thresholds. Panel b plots the changes in Weber fraction, as in panel a, but as a function of the threshold and surround suppression, averaged across exponentials. Panel c plots the change in Weber fraction, as in panel a, but as a function of the exponential and threshold, averaged across surround suppression. While the parameters had a small effect on the change in Weber fraction (note the narrow range in the color bar), all combinations resulted in a slightly smaller Weber fraction for the 20 vs. 2 deg target. Panel d summarizes the results by plotting the relative frequency of the measured change in Weber fractions across all parameter combinations for simulations with (blue) and without (orange) noise in the motion reliability pathway. Without noise, the Weber fractions modestly decreased from the 2 deg target to the 20 deg target. Across parameter values, the amount of change was too small to match the range of changes we observed in our behavioral data (vertical dashed lines). With noise, the change in Weber fractions matched the range of changes we observed in behavioral data. Overall, the results demonstrate that no combination of model MT neuron parameters allowed circuit behavior to match the behavioral results without the addition of gain noise. Source data are provided as a Source Data file.

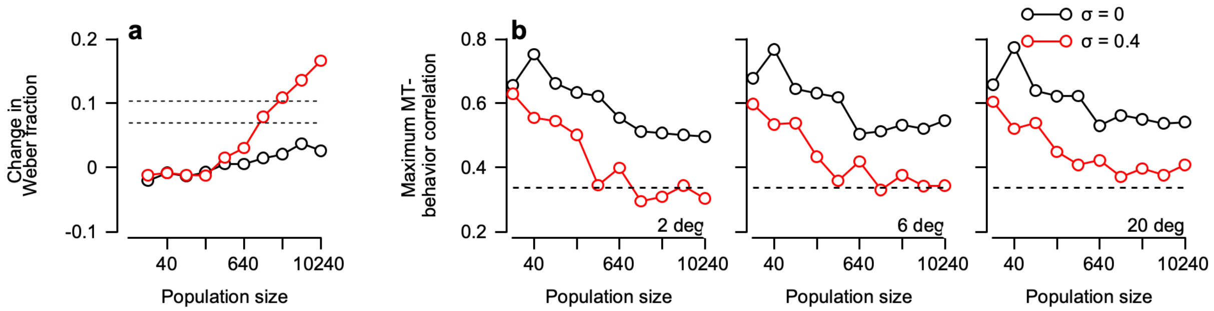

Supplementary Figure 6. Effect of population size on circuit model results. To determine how population size impacts decoding results, we simulated circuit output with model MT neuron populations sizes of 20, 40, 80, 160, 320, 640, 1280, 2560, 5120, or 10240 with (red) or without (black) gain noise. Panel a plots the change in Weber fraction, measured as in Supplementary Figure 4, for each simulation. Only for the smallest population sizes did gain noise fail to produce a change in Weber fraction, and a change large enough to explain the ranges of changes in our behavioral data (dashed lines) was never observed for models without gain noise. We then measured the magnitude of model MT-behavior correlations from the maximum observed MT-correlation across target speeds and model neurons. Panel b plots the magnitude for each population size for the 2 (left), 6 (middle), and 20 (right) deg targets. The magnitude of MT-behavior correlations measured from the circuit model were consistently larger than those observed in physiology (horizontal dashed line) when simulated without gain noise (black), regardless of the population size. Addition of gain noise decreased MT-behavior correlations to realistic levels for moderate to large population sizes (red). Source data are provided as a Source Data file.

Monkey	Direction	Model	Size	w_s	σ	w_m	RMSE	R^2
R	R	w_s fixed		0.12409	0	0	0.45826	0.70777
		w_s flexible					0.21902	0.82111
			2 deg	0.19717	-	0		
			6 deg	0.14373	-	0		
			20 deg	0.11113	-	0		
		Gain noise		0.091969	0.049723	0	0.31905	0.75992
		w_m included		0.064971	0.049828	0.065093	0.31905	0.75992
R	L	w_s fixed		0.12997	0	0	0.35051	0.65139
		w_s flexible					0.21298	0.74678
			2 deg	0.20887	-	0		
			6 deg	0.14952	-	0		
			20 deg	0.11153	-	0		
		Gain noise		0.094232	0.043106	0	0.32536	0.90284
		w_m included		0.066607	0.043201	0.066657	0.32536	0.90284
X	R	w_s fixed		0.10107	0	0	0.63171	0.15946
		w_s flexible					0.27233	0.77305
			2 deg	0.15312	-	0		
			6 deg	0.11507	-	0		
			20 deg	0.083344	-	0		
		Gain noise		0.043282	0.066905	0	0.44069	0.57233
		w_m included		0.030597	0.066936	0.030614	0.44069	0.57233
X	L	w_s fixed		0.11671	0	0	0.89341	0.013785
		w_s flexible					0.76382	0.32342
			2 deg	0.18994	-	0		
			6 deg	0.13868	-	0		
			20 deg	0.086517	-	0		
		Gain noise		0.060075	0.070317	0	0.55503	0.38512
		w_m included		0.042466	0.070381	0.042493	0.55503	0.38512

Supplementary Table 1. Model parameters and fit quality for each monkey and pursuit direction. RMSE and R^2 were calculated from the model fit to target speeds of 4, 12, and 20 deg and the mean and variance of pursuit in response to the 8 and 16 deg/s targets (see Methods).

Supplementary Notes

Supplementary Note 1. Mean and variance of simple gain-noise model

In the main text we consider a model of motor output, m, that applies a sensory-motor gain, G, to an average stimulus estimate, $\hat{s}$ according to:

$$m = (G + \eta_G)(\hat{s} + \eta_s) + \eta_m. \tag{A.1}$$

Variation in the model stems from estimation noise η_s, gain noise η_G, and motor noise η_m. Each noise source is distributed as a zero mean Gaussian with variance σ_s^2, σ_G^2, and σ_m^2 for estimation, gain, and motor noise, respectively. The mean motor output, μ, is defined as

$$\mu = \langle m \rangle = \langle (G + \eta_G)(\hat{s} + \eta_s) + \eta_m \rangle, \tag{A.2}$$

where angled brackets indicate the mean over trials. By expanding and moving deterministic terms outside of the averaging operations, the average can be written as

$$\mu = G\hat{s} + \hat{s}\langle \eta_G \rangle + G\langle \eta_s \rangle + \langle \eta_m \rangle + \langle \eta_G \eta_s \rangle. \tag{A.3}$$

Because they are defined as zero mean stochastic variables, $\langle \eta_G \rangle$, $\langle \eta_s \rangle$, and $\langle \eta_m \rangle$ are all zero. Because η_G and η_s are independent, their joint probability distribution, $p(\eta_G, \eta_s)$, can be written as $p(\eta_G)p(\eta_s)$, and $\langle \eta_G \eta_s \rangle = \int \eta_G p(\eta_G) \int \eta_s p(\eta_s) d\eta_s d\eta_G$, which equals $\langle \eta_G \rangle \langle \eta_s \rangle$. Therefore, the final term on the right hand side of equation A.3 is also zero, and

$$\mu = G\hat{s}. \tag{A.4}$$

The variance of this sensory-motor process, VAR, is defined as

$$\text{VAR} = \langle [m - \mu]^2 \rangle, \tag{A.5}$$

where angled brackets again correspond to averaging over trials. Substituting equations A.1 and A.4, we have

$$\text{VAR} = \langle [(G + \eta_G)(\hat{s} + \eta_s) + \eta_m - G\hat{s}]^2 \rangle. \tag{A.6}$$

Expanding and moving the deterministic variables outside of the averaging operations gives us

$$\text{VAR} = \hat{s}^2 \langle \eta_G^2 \rangle + G^2 \langle \eta_s^2 \rangle + \langle \eta_m^2 \rangle + \langle \eta_G^2 \eta_s^2 \rangle + 2G\hat{s}\langle \eta_G \eta_s \rangle + 2\hat{s}\langle \eta_G^2 \eta_s \rangle + 2G\langle \eta_G \eta_s^2 \rangle + 2G\langle \eta_s \eta_m \rangle + 2\hat{s}\langle \eta_G \eta_m \rangle + 2\langle \eta_G \eta_s \eta_m \rangle. \tag{A.7}$$

Because η_G, η_s, and η_m are zero mean, $\langle \eta_G^2 \rangle = \sigma_G^2$, $\langle \eta_s^2 \rangle = \sigma_s^2$, and $\langle \eta_m^2 \rangle = \sigma_m^2$. When the variables η_G and η_s are independent, $\langle \eta_G^2 \eta_s^2 \rangle = \int \eta_G^2 p(\eta_G) \int \eta_s^2 p(\eta_s) d\eta_s d\eta_G$, which simplifies to $\sigma_G^2 \sigma_s^2$. As above, the remaining terms average to zero. This leaves

$$\text{VAR} = \sigma_G^2 \hat{s}^2 + w_s^2 G^2 \hat{s}^2 + \sigma_G^2 w_s^2 \hat{s}^2 + w_m^2 \mu^2, \tag{A.8}$$

where we substitute $w_s^2 \hat{s}^2$ for σ_s^2 following standard models of signal-dependent noise in sensory processing, and $w_m^2 \mu^2$ for σ_m^2 following standard models of signal-dependent noise in motor processing. Rearranging and substituting μ, we arrive at the definition of motor variance in the main text

$$\text{VAR} = (w_s^2 + w_m^2)\mu^2 + (\sigma_G^2 + \sigma_G^2 w_s^2)\frac{\mu^2}{G^2}. \tag{A.9}$$

Supplementary Note 2. Nonlinearities in sensory estimation

The results in section A.1 apply broadly to any estimator of a stimulus, s, where the variation in estimation can be modeled as depending on the estimate, $\hat{s}$. However, a more realistic model of sensory estimation applies an estimator to a noisy sensory measurement. This can be modeled as $\hat{s} = f[s + \eta_s]$, where f is a generally nonlinear function, s is the stimulus, and η_s is additive noise in the measurement process. We can then model motor output, m, after application of sensory-motor gain, G, as

$$m = (G + \eta_G)(f[s + \eta_s]). \tag{A.10}$$

As in section A.1, η_G models noise in the gain process and both η_G and η_s are zero mean with variance σ_G^2 and σ_s^2, respectively. If we assume independence of the stochastic variables, the average motor output, μ, will be

$$\mu = \langle Gf[s+\eta_s]\rangle. \tag{A.11}$$

If η_s is small, then $f[s+\eta_s] = f[s] + \frac{df}{ds}|_s\eta_s$ and

$$\mu = Gf[s]. \tag{A.12}$$

As above, the variance in sensory-motor output is defined as

$$\mathrm{VAR} = \langle(m-\mu)^2\rangle. \tag{A.13}$$

Substituting equations A.10 and A.12, expanding and simplifying, we arrive at

$$\mathrm{VAR} = \sigma_G^2 f[s]^2 + (G^2+\sigma_G^2)w_s^2 s^2\left(\frac{df}{ds}|_s\right)^2, \tag{A.14}$$

where we have made use of the fact that the variance in sensory noise scales with the stimulus according to $w_s^2 s^2$, as in section A.1. Rearranging and substituting μ, we have

$$\mathrm{VAR} = w_s^2 G^2 s^2\left(\frac{df}{ds}|_s\right)^2 + \sigma_G^2 w_s^2 s^2\left(\frac{df}{ds}|_s\right)^2 + \frac{\sigma_G^2}{G^2}\mu^2. \tag{A.15}$$

Here we see that variance will be partitioned into three components: (1) a partition related to gain noise scaled by the mean output, μ; (2) a partition that scales with $G^2 s^2$ according to w_s; and (3) a partition representing the mixture of noise sources, σ_G and w_s, scaled with s. Most important to the results here, partition (1) retains the inverse relationship of its contribution to the variance and the mean gain, G, extending the result of section A.1 to the more realistic case of sensory noise followed by a potentially nonlinear estimation process. However, it is important to note that partitions (2) and (3) will depend on the derivative of f w.r.t. s evaluated at s and the results are more complicated in general (see Supplementary Figure 2). Using this formulation, it can be easily shown that, when the estimator is linear, estimation noise from section A.1 is equivalent to sensory noise with the slope of the estimator absorbed into w_s.

Finally, we note that, for simplicity, we have dropped the addition of a motor noise source (e.g. η_m in section A.1). Inclusion of motor noise simply adds to the variance as in equation A.8.

References

1. Jazayeri, M. & Shadlen, M. N. Temporal context calibrates interval timing. *Nat. Neurosci.* **13**, 1020–1026 (2010).

2. Cicchini, G. M., Arrighi, R., Cecchetti, L., Giusti, M. & Burr, D. C. Optimal encoding of interval timing in expert percussionists. *J. Neurosci.* **32**, 1056–1060 (2012).

3. Egger, S. W. & Jazayeri, M. A nonlinear updating algorithm captures suboptimal inference in the presence of signal-dependent noise. *Sci. Rep.* **8**, 12597 (2018).
