# Supplementary material for: Neural structure of a sensory decoder for motor control
Source: Nat Commun. 2022 Apr 5;13:1829. doi: 10.1038/s41467-022-29457-4 (PMC8983777; doi:10.1038/s41467-022-29457-4)
Supplement: Supplementary file 3 — Reporting Summary [file 41467_2022_29457_MOESM3_ESM.pdf]

nature research

Corresponding author(s): Seth W. Egger

Last updated by author(s): Feb 10, 2022

Reporting Summary

Nature Research wishes to improve the reproducibility of the work that we publish. This form provides structure for consistency and transparency in reporting. For further information on Nature Research policies, see our Editorial Policies and the Editorial Policy Checklist.

Statistics

For all statistical analyses, confirm that the following items are present in the figure legend, table legend, main text, or Methods section.

n/a	Confirmed	
☐	☒	The exact sample size (*n*) for each experimental group/condition, given as a discrete number and unit of measurement
☐	☒	A statement on whether measurements were taken from distinct samples or whether the same sample was measured repeatedly
☐	☒	The statistical test(s) used AND whether they are one- or two-sided<br>*Only common tests should be described solely by name; describe more complex techniques in the Methods section.*
☒	☐	A description of all covariates tested
☐	☒	A description of any assumptions or corrections, such as tests of normality and adjustment for multiple comparisons
☐	☒	A full description of the statistical parameters including central tendency (e.g. means) or other basic estimates (e.g. regression coefficient) AND variation (e.g. standard deviation) or associated estimates of uncertainty (e.g. confidence intervals)
☐	☒	For null hypothesis testing, the test statistic (e.g. *F*, *t*, *r*) with confidence intervals, effect sizes, degrees of freedom and *P* value noted<br>*Give P values as exact values whenever suitable.*
☒	☐	For Bayesian analysis, information on the choice of priors and Markov chain Monte Carlo settings
☒	☐	For hierarchical and complex designs, identification of the appropriate level for tests and full reporting of outcomes
☒	☐	Estimates of effect sizes (e.g. Cohen's *d*, Pearson's *r*), indicating how they were calculated

Our web collection on statistics for biologists contains articles on many of the points above.

Software and code

Policy information about availability of computer code

Data collection	Data was collected using custom software (Maestro v4.0.0; available for free at https://sites.google.com/a/srscicomp.com/maestro/home)
Data analysis	Data was analyzed using custom code developed in Matlab (R2017a and R2020b). Code for data analysis and implementation of our biomimetic circuit is available at https://doi.org/10.5281/zenodo.5941607.

For manuscripts utilizing custom algorithms or software that are central to the research but not yet described in published literature, software must be made available to editors and reviewers. We strongly encourage code deposition in a community repository (e.g. GitHub). See the Nature Research guidelines for submitting code & software for further information.

Data

Policy information about availability of data

All manuscripts must include a data availability statement. This statement should provide the following information, where applicable:

- Accession codes, unique identifiers, or web links for publicly available datasets
- A list of figures that have associated raw data
- A description of any restrictions on data availability

All data associated with this study are available at https://doi.org/10.5281/zenodo.5889167. Source data are provided with this paper.

Field-specific reporting

Please select the one below that is the best fit for your research. If you are not sure, read the appropriate sections before making your selection.

☒ Life sciences ☐ Behavioural & social sciences ☐ Ecological, evolutionary & environmental sciences

For a reference copy of the document with all sections, see nature.com/documents/nr-reporting-summary-flat.pdf

Life sciences study design

All studies must disclose on these points even when the disclosure is negative.

Sample size	Trial numbers where selected based mainly on our experience in previous experiments that found accurate estimates of mean and variance with 30 trials per condition (e.g. Osborne et. al., 2007). Pilot experiments performed by SWE with different behavioral tasks found that reproducible measurements of the mean and variance of pursuit initiation could be achieved with approximately 50 trials per behavioral condition. Repeated simulations of our 'gain noise' model confirmed that robust estimates of the mean and variance of pursuit initiation could be found from approximately 50 trials per condition. Performing the experiment with the first subject (monkey R) with about 130 trials per condition allowed us to confirm our initial estimate.
Data exclusions	Data for trials in which a saccade occurred within the window of analysis (0 to 250 ms from motion onset) was excluded. Saccades prevent the accurate measurement of smooth pursuit responses necessary to test the relationship between variance and mean in behavior.
Replication	Reproducibility of our findings was achieved by replicating the results in two different directions of pursuit and using a second experimental animal. Replication was successful in all cases.
Randomization	Our experiment did not involve allocation to experimental and control groups. Each animal experience all experimental conditions, which where selected psuedorandomly.
Blinding	Subjects were not allocated to different groups, and therefore blinding was not relevant to our study.

Reporting for specific materials, systems and methods

We require information from authors about some types of materials, experimental systems and methods used in many studies. Here, indicate whether each material, system or method listed is relevant to your study. If you are not sure if a list item applies to your research, read the appropriate section before selecting a response.

Materials & experimental systems

n/a	Involved in the study	
☒	☐	Antibodies
☒	☐	Eukaryotic cell lines
☒	☐	Palaeontology and archaeology
☐	☒	Animals and other organisms
☒	☐	Human research participants
☒	☐	Clinical data
☒	☐	Dual use research of concern

Methods

n/a	Involved in the study	
☒	☐	ChIP-seq
☒	☐	Flow cytometry
☒	☐	MRI-based neuroimaging

Animals and other organisms

Policy information about studies involving animals; ARRIVE guidelines recommended for reporting animal research

Laboratory animals	Two male rhesus macaques (Macaca mullata) aged 12 and 14 years were used in our experiments.
Wild animals	The study did not involve wild animals.
Field-collected samples	The study did not collect field samples.
Ethics oversight	All experimental protocols were approved by the Institutional Animal Care and Use Committee at Duke University (A085-18-04) and performed in accordance with the National Institutes of Health Guide for the Care and Use of Laboratory Animals.

Note that full information on the approval of the study protocol must also be provided in the manuscript.
